# Lactate shuttling as an allostatic means of thermoregulation in the brain

**DOI:** 10.3389/fnins.2023.1144639

**Published:** 2023-05-12

**Authors:** Daniel A. Kane, Alexander C. Y. Foo, Erin B. Noftall, Karen Brebner, D. Gerrard Marangoni

**Affiliations:** ^1^Department of Human Kinetics, St. Francis Xavier University, Antigonish, NS, Canada; ^2^Department of Chemistry, St. Francis Xavier University, Antigonish, NS, Canada; ^3^Department of Psychology, St. Francis Xavier University, Antigonish, NS, Canada

**Keywords:** bioenergetics, brain, calorimetry, lactate, mitochondria, pyruvate, thermal physiology

## Abstract

Lactate, the redox-balanced end product of glycolysis, travels within and between cells to fulfill an array of physiologic functions. While evidence for the centrality of this lactate shuttling in mammalian metabolism continues to mount, its application to physical bioenergetics remains underexplored. Lactate represents a metabolic “cul-de-sac,” as it can only re-enter metabolism by first being converted back to pyruvate by lactate dehydrogenase (LDH). Given the differential distribution of lactate producing/consuming tissues during metabolic stresses (e.g., exercise), we hypothesize that lactate shuttling vis-à-vis the exchange of extracellular lactate between tissues serves a thermoregulatory function, i.e., an allostatic strategy to mitigate the consequences of elevated metabolic heat. To explore this idea, the rates of heat and respiratory oxygen consumption in saponin-permeabilized rat cortical brain samples fed lactate or pyruvate were measured. Heat and respiratory oxygen consumption rates, and calorespirometric ratios were lower during lactate vs. pyruvate-linked respiration. These results support the hypothesis of allostatic thermoregulation in the brain with lactate.

## Introduction

In mammals, most of the energy used to sustain life is derived from the oxidation of fatty acids, amino acids, and glucose by oxygen (O_2_) to form water and CO_2_ in the mitochondria. Because these oxidation reactions are exothermic, an organism will generate heat in proportion to its metabolic rate (Silva, 2006). Metabolic heat from aerobic respiration contributes to the maintenance of body temperature within a relatively narrow range (homeothermic endothermy) that is among the hallmarks of mammalian evolution (Crompton et al., 1978; Silva, 2006). However, during periods of elevated whole-body metabolic activity associated with intense or prolonged physical activity, this excess heat can contribute to the development of hyperthermia, necessitating the evolution of various heat-dissipation mechanisms to maintain thermal homeostasis (Brotherhood, 2008). In peripheral tissues such as skeletal muscles, metabolic heat can be dissipated through surface radiation and evaporative cooling (i.e., sweating), mediated largely by vascular convective heat transfer (González-Alonso, 2012). The more spherical geometry of the brain and its location within the cranial cavity makes it less conducive to dissipating heat via transcranial mechanisms (Nelson and Nunneley, 1998; Wang et al., 2014). Cerebral blood flow and volume are therefore considered the primary means of thermal regulation in the human brain (Nybo, 2012; Wang et al., 2014). However, the relatively small temperature gradient between the body core and the brain may limit the efficacy of this heat transfer mechanism during exercise in hot environments, especially in species like humans and rats which lack a carotid rete and the selective brain cooling it affords some other animals (Nybo, 2012). While some cooling is possible as blood travels along the carotid artery in humans, the transit time may be too short to allow for sufficient heat removal, even when artificial cooling is applied externally (Nybo, 2012). This lack of cooling is puzzling given that the brain is responsible for some 20% of the total oxygen consumption in humans at rest, despite constituting only 2% of body mass (Rolfe and Brown, 1997). Additionally, the brain has been shown to be very sensitive to changes in temperature; brain hyperthermia can be induced through a variety of physiological stressors such as bacterial infection/inflammation (Thompson et al., 2003) or recreational drug use (Brown and Kiyatkin, 2004; Kiyatkin et al., 2014), and has been implicated in the negative health outcomes associated with these conditions. At the same time, limiting oxygen to the brain for aerobic respiration quickly leads to brain damage or death of the organism (Sarkar et al., 2019). Given these considerations, understanding the mechanisms by which cerebral temperatures can be regulated is of particular concern to the field of neuroscience.

Some insight can be gleaned by considering the metabolic behavior of the brain itself. Conventional wisdom posits that the brain is an obligate glucose consumer under normal resting conditions (e.g., Hui et al., 2017; Dienel, 2019a). The human brain may release a small amount of lactate at rest, but during exercise, cerebral lactate uptake increases in proportion to the rise in arterial lactate concentration (Quistorff et al., 2008; Siebenmann et al., 2021), contributing to 27.8% of cerebral metabolic rate during maximal exercise (Smith and Ainslie, 2017). Thus, evidence generally supports that the brain possesses a substantial capacity to use lactate as a fuel (e.g., Smith et al., 2003; Gallagher et al., 2009; van Hall et al., 2009; Boumezbeur et al., 2010; Wyss et al., 2011; and as reviewed in Schurr, 2006). Anticipatory regulation [i.e., *allostasis*; (Sterling and Eyer, 1988)] is a logical strategy to guard against threats to homeostatic brain and body temperatures (Sterling, 2012). Mason (2017) convincingly argued a role for lactate and lactate shuttling in the context of neuroenergetic allostasis, allostatic load, and allostatic overload associated with injury and illness. Given the brain’s capacity to consume lactate, and the conditions in which muscle heat production and lactate levels increase (i.e., exercise), one might wonder whether lactate shuttling constitutes an allostatic means of mitigating cerebral metabolic heat production during intense or prolonged exercise in hot environments.

The early comment by Fletcher and Hopkins (1907) that “there is hardly any important fact concerning the lactic acid formation in muscle which, advanced by one observer, has not been contradicted by some other” echoes across the literature. Indeed, use of lactate as a fuel has largely supplanted its former reputation as a “dead-end” consequence of anaerobic metabolism and glycogenic precursor; and yet, prior misinterpretations of lactate metabolism carry an historical inertia against which evolving understandings must contend (e.g., Schurr, 2017; Brooks, 2018; Ferguson et al., 2018). In 1923, A.V. Hill and Otto Meyerhof split the Nobel Prize in Physiology or Medicine for discoveries relating to the production of heat in muscle (Hill), and the relationship between oxygen consumption and lactate metabolism in muscle (Meyerhof) (Barclay and Curtin, 2022). Hill’s work, carried out in isolated frog muscle, indicated that consumption of lactate by muscle cells yielded significantly less heat than would be expected for the complete combustion (oxidation) of lactate (Schurr, 2017). As noted by Schurr (2017), the prevailing interpretation of these results at the time was that most of the lactate produced during muscle contractions is utilized for glycogen resynthesis, with a minor fraction undergoing complete oxidation. This view has been largely revised for mammalian lactate metabolism vis-à-vis lactate shuttling (Brooks and Gaesser, 1980; Gaesser and Brooks, 1980; Miller et al., 2002a,b). As first conceived by Brooks (1985), a central tenet of the lactate shuttle hypothesis is that lactate travels within and between cells as an oxidizable fuel (Brooks, 2020). While evidence for the centrality of this concept of lactate-as-fuel in mammalian metabolism continues to mount (e.g., Hui et al., 2017), and the array of physiologic functions ascribed to lactate has only expanded (Brooks et al., 2022a), research examining the thermal bioenergetics of lactate oxidation remains comparatively sparse since the time of Hill and Meyerhof. The lactate shuttle may provide a pathway by which the “missing” heat noted by Hill may be reconciled by shuttling lactate out of the muscle and into the blood stream in the intact organism (Bassett Jr, 2002).

**Figure 1:**
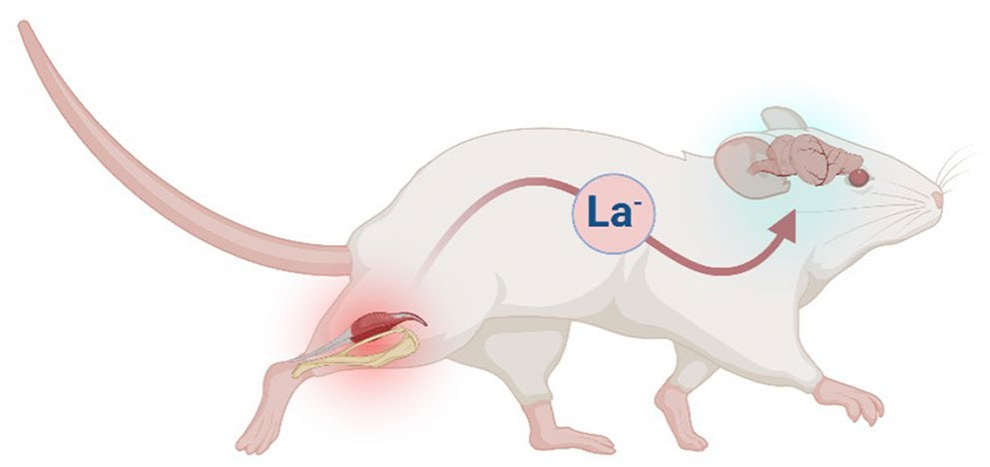
Hypothetical model in which the lactate shuttle serves a thermoregulatory function in mammals. In the example depicted (mouse), excess lactate (La^−^) released by skeletal muscle during periods of elevated glycolysis (e.g., exercise) is shuttled to the brain via circulating blood. In the model, metabolism of La^−^ of extracellular origin is accompanied by a lower rate of heat production. Figure created with BioRender.com.

The present exploration is informed by two foundational assumptions: (1) lactate is effectively always the end-product of glycolysis (Rogatzki et al., 2015; Schurr, 2017); and (2) lactate diffuses across cell membranes via monocarboxylate transporters (MCTs) in proportion to its concentration gradient (Hertz and Dienel, 2005). Thus, we wondered if a thermoregulatory function might be ascribed to the metabolism of lactate, and measured rates of heat production and oxygen consumption in permeabilized samples of rat brain respiring with lactate versus pyruvate (+NAD^+^). The hypothesis being tested is summarized in Figure 1 and Tables 1, 2.

**Table 1:** Standard enthalpies of formation (Δ_f_*H*°) and ionization (Δ_ion_*H*°) associated with complete lactate and pyruvate oxidation, as reported in Miller and Smith-Magowan (1990), Goldberg et al. (2002), and Hammes and Hammes-Schiffer (2015).

	Δ_f_*H*° (kJ mol^−1^)	Δ_ion_*H*° (kJ mol^−1^)
Glucose (aq)	−1267.11	
HCO_3_^−^ (aq)	−689.93	
CO_2_ (aq)	−413.26	
H_2_O (l)	−285.83	
NAD^+^	−10.26	
NADH	−41.38	
Pyruvic acid (aq)	−603.7	+12.13
Lactic acid (aq)	−686.2	−0.43
HEPES (aq)		+20.4
H_2_CO_3_ (aq)		+9.15

**Table 2:** Calculated standard reaction enthalpy changes (Δ*H*°) and oxycaloric ratios (OCR) for reactions in skeletal muscle and brain.

Reaction	Δ*H*° (kJ mol^−1^)	OCR (kJ mol^−1^ O_2_)
*In vivo*		
Glucose (aq) + 2HCO_3_^−^ (aq) ⇋ 2 lactate (aq) + 2CO_2_ (aq) + 2H_2_O (l)	−124.47	
Lactate (aq) + 3O_2_ (g) → HCO_3_^−^ (aq) + 2CO_2_ (aq) + 2H_2_O (l)	−1401.48	467.16
Lactate (aq) + NAD^+^ (aq) + HCO_3_^−^ (aq) ⇋ Pyruvate (aq) + NADH (aq) + CO_2_ (aq) + H_2_O (l)	+45.63	
Lactate (aq) + 0.5O_2_ (g) ⇋ Pyruvate (aq) + H_2_O (l)	−190.77	381.54
Pyruvate + 2.5O_2_ (g) → HCO_3_^−^ (aq) + 2CO_2_ (aq) + H_2_O (l)	−1210.71	484.28
*Experimental* ^a^		
Lactate (aq) + NAD^+^ (aq) + HEPES^−^ ⇋ Pyruvate (aq) + NADH (aq) + HEPES	+43.54	

a Reaction with excess NAD^+^.

Scott and Kemp (2005) tested whether mitochondrial lactate oxidation could function as a heat sink in the manner described above. Using calorimetry and oxygen consumption measurements in parallel, they determined the calorespirometric (CR) ratio (kJ mol^−1^ O_2_) during either lactate- or pyruvate-supported respiration in permeabilized pEF TB/C3 hybridoma cells and mouse myocardial fibers. They found the CR ratio with lactate was the same as that with pyruvate (Scott and Kemp, 2005). Unfortunately, they did not report either heat or oxygen consumption rates. Also, Scott and Kemp (2005) did not include the cofactor for the L-lactate dehydrogenase (LDH) reaction, NAD^+^, the non-mitochondrial matrix portions of which may be lost with permeabilized tissues and cells. Here, we measured rates of heat and oxygen consumption in permeabilized samples of rat brain respiring with lactate versus pyruvate (+NAD^+^). In contrast to Scott and Kemp (2005), we find that the CR ratio is lower in brain tissue respiring with lactate. These results are discussed from both mechanistic and teleological perspectives.

## Methods

### Animals and reagents

Six male Wistar rats were purchased from Charles River Laboratories (Montreal, Canada) and acclimated for two weeks in an on-site facility with temperature control and reversed light:dark cycle prior to experiments. These rats were doubly housed in cages with access to running wheels, standard chow, and water *ad libitum*. On the day of the experiment, the rats were weighed (average body mass: 331 ± 36 g), and anesthetized via an intraperitoneal injection of 250 mg/ml sodium pentobarbital (Vetoquinol, QC, Canada). All other chemicals, including sodium L-lactate and sodium pyruvate were purchased from MilliporeSigma. Under deep anesthesia, the rats were decapitated, and their brains immediately dissected using blade and large rat brain anatomical matrix (1.0 mm Coronal sections, Zivic Instruments, Pittsburgh, United States). Dissected tissues were prepared for analysis as previously described (Herbst and Holloway, 2015; Herbst et al., 2018). All procedures in this study were approved by the St. Francis Xavier University Animal Care Committee, and conformed to the standards of the Canadian Council on Animal Care.

### Heat production and oxygen consumption rate measurements

Following dissection, small (<35 mg) samples of frontal cortex were weighed, and then added to either the isothermal titration microcalorimeter (ITC; Model 4,200, Calorimetry Sciences Corporation), or the Oxygraph O2k high-resolution respirometer (O2k; OROBOROS Instruments, Innsbruck). The chambers of both instruments contained 1 ml (ITC) or 1.5 ml (O2k) of MiR05 mitochondrial respiration buffer (Pesta and Gnaiger, 2012), to which the following had been added: 20 mM creatine, 50 μg/mL saponin, 4 mM malate, 2 mM NAD^+^, and 5 mM ADP. The ITC and O2k protocols were performed in parallel, at 25°C, and with continual stirring. Following relative stabilization of the ITC heat rate trace, either L-lactate or pyruvate (10 mM final concentration) was injected into the chambers of each instrument. Due to the time required for the ITC to stabilize, measurements were made at 25°C (as opposed to 37°C, for example) and only one experimental substrate could reliably be tested per sample, due to inevitable oxygen limitations apparent at the end of each trial, as indicated in the O2k. Heat rate (d*Q*/d*t*) data were expressed as μJ s^−1^ mg^−1^ wet weight. Respiratory oxygen consumption rate (*J*O_2_) data were expressed as pmol s^−1^ mg^−1^ wet weight. dQ/dt and *J*O_2_ data were processed in parallel to give the calorespirometric (CR) ratio (kJ mol^−1^ O_2_), according to Skolik et al. (2018).

### Analysis

Data are mean ± SEM and analyzed using Graphpad Prism (version 9.5.0) software. d*Q*/d*t*, *J*O_2_, and the CR ratio with lactate were compared to those with pyruvate using independent student’s *t*-test (α-level = 0.05).

## Results

To explore the hypothesis that the metabolic heat associated with lactate versus pyruvate oxidation differs, the rates of heat (d*Q*/d*t*) and respiratory oxygen consumption (*J*O_2_) in saponin-permeabilized rat cortical brain samples fed lactate or pyruvate were measured.

**Figure 2:**
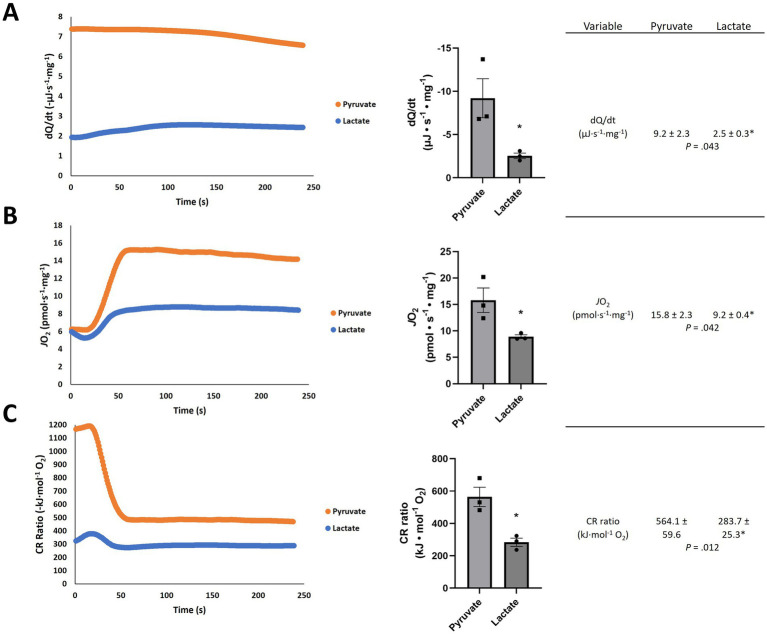
Representative traces of **(A)** heat rate (d*Q*/d*t*), **(B)** oxygen consumption rate (*J*O_2_) and **(C)** calorespirometric (CR) ratio in permeabilized rat frontal cortex samples fed pyruvate or lactate + NAD^+^. Numeric values are mean ± SEM; *n* = 3; *p-values from *t*-tests with 6 independent trials.

The results in Figure 2 show the d*Q*/d*t*, *J*O_2_, and CR ratio in brain tissue are all significantly lower in the presence of excess lactate from an external source. The d*Q*/d*t* with lactate (Figure 2A) is lower by about a factor of 3 and the *J*O_2_ is lower by about a factor of 2 compared with the experiment with pyruvate. The measured CR ratio for pyruvate (564.1 ± 59.6 kJ mol^−1^ O_2_; Figure 2) is in reasonable agreement with its oxycaloric equivalent (484.28 kJ mol^−1^ O_2_; Table 2), but the CR ratio for lactate is markedly lower (283.7 ± 25.3 kJ mol^−1^ O_2_; Figure 2) than its oxycaloric equivalent (467.16 kJ mol^−1^ O_2_; Table 2). However, we must consider: is a similar reduction in metabolic heat rate likely to occur in a living organism, or is this an artefact of the experimental conditions? The agreement between our pyruvate experimental values and both the theoretical oxycaloric equivalent and other literature sources suggest that this is not the case.

## Discussion

In the present exploration, we hypothesized a lower rate of heat production associated with mitochondrial lactate vs. pyruvate oxidation in the rat brain, reflective of a larger working model in which the metabolism of lactate serves a thermoregulatory function (Figure 1). Addition of lactate elicited lower d*Q*/d*t* and *J*O_2_ per mg of rat tissue when compared to an equimolar addition of pyruvate (Figure 2). Interestingly, the CR ratio with lactate (283.7 ± 25.3 kJ mol^−1^ O_2_) fell below both the theoretical oxycaloric equivalent predicted from Tables 1, 2 (467.16 kJ mol^−1^ O_2_) and that predicted by Thornton’s rule [455 ± 15 kJ mol^−1^ O_2_ (Hansen et al., 2004)].

Thornton’s Rule states that the heat of combustion of organic compounds per mole of O_2_ is relatively constant (Thornton, 1917). This rule implies that as a cell system moves toward 100% aerobic metabolism, the CR ratio will approach the theoretical oxycaloric constant for the particular cell line or tissue type and substrate source (Skolik et al., 2018); published oxycaloric constants therefore tend to range between 430 to 480 kJ mol^−1^ (Gnaiger and Kemp, 1990; Kemp, 2000). In the context of calorespirometry in a cell catabolizing glucose under aerobic conditions, a CR ratio below the oxycaloric equivalent implies the products of glycolysis (i.e., lactate and/or pyruvate) are not fully oxidized to CO_2_ and water.

There are two ways to decrease metabolic heat rate: decrease the rate of the reaction(s) producing the heat, and/or alter the chemistry (e.g., by changing the conditions in which the reaction(s) occur). In our experiments, the O_2_ consumption rate is a direct measure of the complete oxidative metabolic rate in the permeabilized brain tissue, and the CR ratio indicates the reactions that are occurring. Using Thornton’s rule (oxycaloric equivalent; Table 2) and the measured O_2_ consumption rates (Figure 2), the predicted heat rate for pyruvate-fed brain tissue is 7.7 μJ s^−1^ mg^−1^ and for lactate-fed brain tissue it is 4.3 μJ s^−1^ mg^−1^. These results alone directly support the hypothesis, inasmuch as the complete oxidation of lactate, and thus predicted rate of heat is lower with lactate than pyruvate. The calculated heat rate and CR ratio for pyruvate agree with the measured heat rate and CR ratio, but the heat rate and CR ratio for lactate are lower than values predicted when applying the oxycaloric ratio (Table 2) to the oxygen consumption rate. To understand this disagreement between the observed and predicted values, and further develop the model, consider that the complete oxidation of lactate can be divided into three reactions, each with calculated standard reaction enthalpies (Δ*H*_n_; n = reaction):


(1) $$\begin{array}{c} {\text{Lactate}}_{\left(\text{aq}\right)}+{\text{NAD}}_{\left(\text{aq}\right)}^{+}+{\text{HCO}}_{3\;\left(\text{aq}\right)}^{-}⇌{\text{Pyruvate}}_{\left(\text{aq}\right)}+{\text{NADH}}_{\left(\text{aq}\right)}\\ \;+{\text{CO}}_{2\;\left(\text{aq}\right)}+H_{2}O_{\left(l\right)}\;\Delta H_{1}=+45.63\;\text{kJ}\;{\text{mol}}^{-1} \end{array}$$

(2) $${\text{Lactate}}_{\left(\text{aq}\right)}+0.5\;O_{2\;\left(g\right)}\to {\text{Pyruvate}}_{\left(\text{aq}\right)}+H_{2}O_{\left(l\right)}\;\;\Delta H_{2}=-190.77\;{\text{kJ mol}}^{-1}$$

(3) $$\begin{array}{c} \text{Pyruvate}+2.5O_{2\;(g)}\to {\text{HCO}}_{3\;(\text{aq})}^{-}+2{\text{CO}}_{2\;(\text{aq})}\\ +H_{2}O_{\left(l\right)}\;\Delta H_{3}=-1210.71\;\text{kJ}\;{\text{mol}}^{-1} \end{array}$$

Combining the three reactions (R), we can produce an overall equation describing the rate of heat generation:


(4) $$\begin{array}{l} dQ/dt=R1\left(+45.63\;{\text{kJ mol}}^{-1}\right)+R2\left(-190.77\;\text{kJ}\;{\text{mol}}^{-1}\right)\\ \;+R3\left(-1210.71\;\text{kJ}\;{\text{mol}}^{-1}\right) \end{array}$$

where (R1), (R2), and (R3) are the rate of reactions (1)–(3), respectively. According to the principles of enzyme kinetics, the rate of reactions (1)–(3) depend on the concentrations of substrates (lactate, pyruvate, or NAD^+^), and the relative activities of LDH and the enzyme(s) that limit the rate of complete pyruvate and NADH oxidation. If the conversion of lactate to pyruvate (R1) is faster than oxidation removes pyruvate (R3), then pyruvate will build up and diffuse out of the cell. This gives rise to a decoupling between the rate of lactate consumption (R1), CO_2_ production (R3), and heat generation (Σ(R1 × Δ*H*_1_) (R2 × ΔH_2_) (R3 × Δ*H*_3_)). However, in the experiments carried out in the present work, if the reaction (R1) occurs outside of the matrix, it becomes:


(5) $$\begin{array}{l} {\text{Lactate}}_{\left(\text{aq}\right)}+{\text{NAD}}_{\left(\text{aq}\right)}^{+}+{\text{HEPES}}_{\left(\text{aq}\right)}^{-}⇌{\text{Pyruvate}}_{\left(\text{aq}\right)}+{\text{NADH}}_{\left(\text{aq}\right)}\\ \;+{\text{HEPES}}_{\left(\text{aq}\right)}\;\;\Delta H_{5}=+43.54\;\text{kJ}\;{\text{mol}}^{-1} \end{array}$$

If we assume negligible NAD^+^ regeneration in the experiment, the combined (R5) and (R3) is:


(6) $$\begin{array}{l} {\text{Lactate}}_{\left(\text{aq}\right)}+{\text{NAD}}_{\left(\text{aq}\right)}^{+}+{\text{HEPES}}_{\left(\text{aq}\right)}^{-}+2.5O_{2\;\left(g\right)}⇌{\text{NADH}}_{\left(\text{aq}\right)}+{\text{HEPES}}_{\left(\text{aq}\right)}\\ \;+{\text{HCO}}_{3\;\left(\text{aq}\right)}^{-}+2{\text{CO}}_{2\;\left(\text{aq}\right)}+H_{2}O_{\left(l\right)}\;\;\Delta H_{6}=-1167.17\;{\text{kJ mol}}^{-1} \end{array}$$

Because both the predicted d*Q*/d*t* (4.3 μJ s^−1^ mg^−1^) from the measured *J*O_2_ via Thornton’s Rule and the oxycaloric equivalent for (R6) (466.87 kJ mol^−1^ O_2_) exceed the measured values for both, incomplete lactate oxidation involving a decoupling of the rates of (R5) from (R3) is a plausible explanation. In the experimental conditions, if lactate is only oxidized by NAD^+^ to pyruvate (R5), but the NADH thus formed is reoxidized by oxygen (R2), the Δ*H* = −190.77 kJ mol^−1^ and the CR ratio is 381.54 kJ mol^−1^ O_2_. This is still well above our measured CR ratio for lactate (283.7 ± 25.3 kJ mol^−1^ O_2_). Therefore, it is reasonable to infer that appreciable lactate oxidation to pyruvate occurred (R5), with attenuated oxidation of the pyruvate (R3) and/or negligible reoxidation of NADH (R2) by oxygen. Assuming no NADH reoxidation in (R5) (i.e., 2.5O_2_ per lactate incompletely oxidized), the observed CR ratio with lactate (283.7 kJ mol^−1^ O_2_), Δ*H*_3_, and Δ*H*_5_ give a ratio of (R5) to (R3) equaling 11.5. In other words, the experimentally observed CR ratio with lactate is explained by a rate of (R5) exceeding (R3) by a factor of 11.5. Note that in the organism, NAD^+^ is only a redox cofactor in the catalysis of lactate oxidation to pyruvate, and the regeneration of NAD^+^ is ultimately due to O_2_ oxidation of NADH (R2) *in vivo*. Therefore, the LDH-catalyzed lactate oxidation to pyruvate *in vivo* (R1) would need to exceed the rates of complete pyruvate oxidation (R3) and NADH oxidation (R2) by a factor of 12.06 for the CR ratio to approach our observed 283.7 kJ mol^−1^ O_2_.

According to our hypothetical model, the d*Q*/d*t* during periods of net lactate oxidation depend on the relative activities of LDH and the enzyme(s) that limit the rate of pyruvate oxidation. If the conversion of lactate to pyruvate is faster than oxidation removes pyruvate, then pyruvate would be expected to build up and diffuse out of the cell. Note that L-lactate is likely a direct and/or indirect inhibitor of pyruvate oxidation (discussed below). Using human erythrocytes, which notably lack mitochondria, Minikami and de Verdier (1976) reported that the reaction catalyzed by LDH is the most enthalpic step in glycolysis. This led them to speculate that pyruvate, by virtue of its permeability to cell membranes, may be involved in the regulation of heat production in cell systems (Minikami and de Verdier, 1976). Here, we are suggesting that regulation of metabolic heat is accomplished via lactate of extracellular origin. However, with regard to the afore mentioned buildup of pyruvate predicted by a decoupling of (R1) and (R3), we are not aware of evidence supporting substantial cerebral pyruvate efflux *in vivo*, save for perhaps with hypoxia (Overgaard et al., 2012) or intravenous infusion of hypertonic sodium lactate (e.g., Bouzat et al., 2014). Another potential explanation for nonoxidative removal of pyruvate would be through carboxylation involving pyruvate carboxylase and/or malic enzyme activities (Hassel, 2000). However, we are unaware of reported enthalpies of pyruvate carboxylation. Investigating the potential role of pyruvate carboxylation in the current model are the anticipated objectives of follow-up work.

The CR ratio for pyruvate in the present work agrees with that found by Scott and Kemp (2005): 502 ± 15 kJ mol^−1^ O_2_ in permeabilized hybridoma cells and 506 ± 47 kJ mol^−1^ O_2_ in permeabilized cardiac muscle fibers. However, our CR ratio for brain tissue respiring with lactate is substantially lower than the Scott and Kemp (2005) values: 517 ± 89 kJ mol^−1^ O_2_ in permeabilized hybridoma cells and 502 ± 15 kJ mol^−1^ O_2_ in permeabilized cardiac muscle fibers for lactate oxidation. Note that both our work and Scott and Kemp (2005) used the same HEPES buffer, so that cannot account for any differences. Scott and Kemp (2005) did not include the cofactor for the LDH reaction, NAD^+^, which may be an issue, as lactate was likely oxidized to pyruvate by the 2 mM of NAD^+^ supplied in the present preparation. This reduces the ΔH from an exothermic −190.77 kJ mol^−1^ (Δ*H*_2_) if lactate were oxidized to pyruvate by O_2_ to an endothermic +43.54 kJ mol^−1^ (Δ*H*_5_). The agreement between the measured CR ratio and the calculated oxycaloric ratio for pyruvate shows that pyruvate was oxidized by oxygen, whereas the discrepancy between the experimental and predicted values for lactate imply that lactate may have been oxidized by NAD^+^ faster in the experiment. In living brain tissue, the d*Q*/d*t* is also expected to be from oxidation of lactate to pyruvate by O_2_ (via NADH). Thus, the measured heat rate in the present experiments may be disregarded when considering the heat rate produced by lactate oxidation to pyruvate in the brains of mammals. Instead, the fastest reaction *in vivo* is expected to be (R2) (${\text{Lactate}}_{\left(\text{aq}\right)}+0.5O_{2\;\left(g\right)}⇌{\text{Pyruvate}}_{\left(\text{aq}\right)}+H_{2}O_{\left(l\right)}$ ); but an obvious consideration *in vivo* is lactate oxidation to pyruvate by NAD^+^. NADH produced by extra-matrix LDH in the oxidation of lactate would likely need to be shuttled into the mitochondria via the malate–aspartate shuttle (McKenna et al., 2006; Gellerich et al., 2012; Kane, 2014), or others for example (Passarella et al., 2021), for the expected heat of combustion with lactate to approach that of pyruvate. In the present experiments, it is doubtful that any substantial contribution by remnants of the malate–aspartate shuttle remains in the permeabilized samples tested.

Incomplete lactate oxidation implies potentially elevated NADH, which calls into question whether neurons or glia are even capable of accommodating such a redox shift under physiologic conditions. Large shifts in the NAD^+^/NADH ratio have been observed in neurons and astrocytes treated with high concentrations (>10 mM) of lactate (Wilhelm and Hirrlinger, 2011; Yang et al., 2014), reflecting elevated NADH, but not necessarily NAD^+^ concentrations in cells with multiple NAD^+^ producing/consuming activities. While the physiological accuracy of extremely large rises in NADH have been questioned (Dienel, 2019b), the observed increases in NADH with high lactate in brain cells suggests a capacity for these cells to accommodate redox shifts associated with incomplete lactate oxidation.

The most straightforward explanation for a low *in vivo* value of the CR ratio for lactate comes from the lactate shuttle. Lactate must first be converted to pyruvate prior to any further transformations. Incomplete lactate oxidation involving attenuated oxidation of the resulting NADH could, in theory, account for lower CR ratios. However, in an *in vivo* scenario in which the NADH formed in the oxidation of lactate to pyruvate is not reoxidized by O_2_ (R1), but pyruvate is completely oxidized (R3) reduces the CR ratio from complete lactate oxidation (Table 2) by only 1.13 kJ mol O_2_^−1^. Alternatively, a scenario in which lactate is only oxidized to pyruvate *in vivo* (R2), instead of to CO_2_ and water (Table 2), but the formed NADH is reoxidized by O_2_, reduces the heat produced by 85.62 kJ mol O_2_^−1^ consumed. And while neuronal Ca^+2^ levels, for example, may alter the activity of the malate–aspartate shuttle, (e.g., Contreras and Satrustegui, 2009; recently reviewed by del Arco et al., 2023), the magnitude of the reduction in lactate CR ratios observed in the current work suggests that pyruvate oxidation was affected.

Interestingly, it was reported recently that lactate partially inhibits mitochondrial respiration in permeabilized cardiac fibers from naked mole rats (Huynh and Pamenter, 2022). The inhibitory effect of lactate decreased respiration with either palmitoyl-carnitine (37%) or pyruvate (20%) (Huynh and Pamenter, 2022). On the other hand, heat acclimation has been found to *increase* the expression of mitochondrial LDH (LDHB; the isoform associated with the oxidation of lactate to pyruvate) in rat heart (Mreisat et al., 2020), which should, paradoxically, lead to increased pyruvate oxidation. In isolated rat brain mitochondria, L-lactate has been observed to partially inhibit (29%) mitochondrial respiration with pyruvate (Ling et al., 2012). This was suggested to possibly be the result of competitive inhibition of mitochondrial pyruvate transport, given lactate may share the same mitochondrial transporter (Ling et al., 2012), but it is noted also that Valenti et al. (2002) reported evidence of mitochondrial lactate/pyruvate antiport in rat heart mitochondria. Related to this, membrane potential-dependent enrichment of lactate in mitochondria was recently observed using a ratiometric lactate sensor (Li et al., 2023). Computational modeling of the pyruvate dehydrogenase (PDH) activity under simulated physiological conditions indicates that PDH is largely regulated through product inhibition by NADH (Jelinek and Moxley, 2021). Depending on the submitochondrial location of LDH and the activity of mitochondrial electron shuttles, the NADH formed in the LDH-catalyzed lactate oxidation to pyruvate may therefore be expected to inhibit PDH. Moreover, L-lactate-mediated release of Mg^+2^ from the endoplasmic reticulum and subsequent mitochondrial uptake was demonstrated in hepatocytes (Daw et al., 2020). Mitochondrial uptake of Mg^+2^ led to a marked inhibition of pyruvate-linked mitochondrial oxygen consumption rates (Daw et al., 2020). With low-concentration saponin-permeabilization of the plasma membrane, both the endoplasmic reticulum and mitochondrial membranes remain intact in muscle fibers (Saks et al., 1998). If a similar phenomenon occurs in our preparations (i.e., L-lactate-mediated inhibition of complete pyruvate oxidation), this could spell an appreciable divergence, or “decoupling,” in rates of LDH-catalyzed lactate oxidation to pyruvate and the complete oxidation of the resulting pyruvate. The net result of L-lactate—mediated inhibition of pyruvate oxidation with or without incompletely oxidized NADH formed via LDH is thus expected to result in a decrease in the CR ratio for lactate. Depending on the level of inhibition of complete pyruvate oxidation, oxidation of lactate to pyruvate would need to exceed the rate of mitochondrial NADH shuttling by a factor of about 11.06 (no inhibition of pyruvate oxidation), 8.11 (Ling et al., 2012), or 9.03 (Huynh and Pamenter, 2022) to reach sub-300 CR ratios *in vivo*.

Another potential factor worth mentioning is the experimental media component, lactobionate. It has been suggested that the 60 mM lactobionate of the standard MiR05 respiratory buffer media could inhibit mitochondrial L-lactate transport into the matrix (Passarella et al., 2014, 2021), which if true, could potentially explain our results. However, NAD^+^-dependent respiration with lactate has been observed in permeabilized brain, and skeletal muscle and cardiac fibers in media with (Jacobs et al., 2013; Herbst et al., 2018; Mahalingam et al., 2023) and without (Elustondo et al., 2013) lactobionate. Moreover, lactate-stimulated respiration in the absence of added NAD^+^ was not observed in a study using media without lactobionate to test permeabilized skeletal and cardiac fibers (Ponsot et al., 2005). In any case, we chose to recapitulate the experimental media conditions of Scott and Kemp (2005), which included 60 mM lactobionate. More recently, Lund et al. (2023) demonstrated the confounding effects of osmotic load and counterion (Na^+^) of sodium L-lactate in mice. We used pH-neutral aqueous sodium L-lactate in the present work but compared this to equimolar (10 mM) pH-neutral aqueous sodium pyruvate. Therefore, any potential osmolarity and/or counterion effects should be similar between the lactate and pyruvate conditions tested in the present work.

It is curious that Scott and Kemp (2005) failed to observe a difference in the CR ratios of permeabilized mouse myocardial fibers using either lactate or pyruvate substrates. In comparing the results from the present study with those of Scott and Kemp (2005) a key difference in experimental design is our inclusion of NAD^+^, and the tissue type investigated. Several previous studies report mitochondrial lactate oxidation in muscle, but only after adding NAD^+^ (Szczesna-Kaczmarek, 1990; Elustondo et al., 2013; Jacobs et al., 2013; Mahalingam et al., 2023). Added NAD^+^ should not be necessary for mitochondrial lactate oxidation if LDH resides in the mitochondrial matrix. The most recent version of the MitoCarta inventory of mitochondrial proteins in mouse and human lists a mitochondrial matrix location for L-lactate dehydrogenase in several tissues; the heart and skeletal muscle are not among them (http://www.broadinstitute.org/mitocarta; Rath et al., 2021). Alternatively, the mitochondrial lactate oxidation complex is proposed to directly couple the endergonic LDH-catalyzed oxidation of lactate to pyruvate outside of the matrix with the exergonic redox change in cytochrome *c* oxidase (complex IV of the respiratory chain) during mitochondrial electron transport (Hashimoto et al., 2006, 2008; see: Figure 1 in Hashimoto and Brooks, 2008). However, such a relationship should not influence the heats of reaction and cannot explain the current results beyond incomplete oxidation. To be clear, neither the physical nor functional submitochondrial location of LDH were explored in the current study. Recent discussion of the topic may be found elsewhere (Fulghum et al., 2019; Borst, 2020; Young et al., 2020; Glancy et al., 2021; Passarella et al., 2021; Brooks et al., 2022b). The fact that complete heats of combustion are released or dissipated as heat (i.e., catabolic steady-state) is bioenergetically independent of subcellular compartmentalization, *per se*. However, if subcellular compartmentalization alters the rates of reactions (e.g., by limiting substrate transport) involved in complete lactate oxidation, or alters the conditions in which the reaction(s) occurs (e.g., different buffering compounds in vs. outside the mitochondrial matrix), this would be expected to influence the heat rate. Therefore, a limitation to the current work is the use of standard enthalpies of formation and ionization to estimate reaction enthalpies, which can vary under non-standard conditions.

From a kinetic perspective, the difference in oxygen consumption rates between lactate and pyruvate suggests a level of regulation warranting further exploration in more physiological/pathophysiological contexts. Testing combined substrates, including lactate and pyruvate together in the ratios they are found *in vivo* for example [e.g., as with traumatic brain injury (Vespa et al., 2012)], may better define or refine the findings and ideas proffered in the current work. We thusly join Schurr (2017) in speculating about the possibility of “…controlled utilization” of lactate. Indeed, our heat and oxygen consumption data with lactate do support a more controlled utilization than equimolar pyruvate, and are hypothesized to contribute to observations in the intact organism (see Teleological Perspective below). Whether, or to what degree these phenomena contribute to physiology *in vivo* requires further research.

## Teleological perspective

While extrapolating thermoregulatory responses in exercising rats to human thermal physiology has its potential limitations (Wanner et al., 2015), it is reasonable to expect that similar physical bioenergetics governs cerebral lactate metabolism in both species. In animals lacking a carotid rete such as rats and humans, there is no evidence for whole brain selective cooling, though whether rats possess regional selective brain cooling is controversial (Jessen, 2001). In exercising rats, coercive exercise to exhaustion in the heat can lead to death, coincident with elevated core body temperature (Fruth and Gisolfi, 1983), and core and/or brain hyperthermia appear to limit voluntary exercise with heat in rats (Fuller et al., 1998).

In humans, brain temperature is predominantly dictated by core body and arterial blood temperature (Nybo, 2012), and during prolonged exercise with hyperthermia, heat release from the brain remains inadequate (Nybo et al., 2002). While cerebral oxygenation due to blood flow increases during intense exercise (compared to rest), the cerebral metabolic ratio of O_2_/[glucose + ½lactate] decreases, a finding which has been interpreted to reflect declining complete oxidation, but not accumulation, of glucose and lactate taken up by the brain (Ide et al., 1999; Dalsgaard et al., 2004). Increasing non-oxidative or incomplete cerebral oxidative metabolism is therefore among the plausible explanations for these observations. Any shift towards incomplete vs. complete oxidative disposal of glucose and/or lactate in the brain would also reduce the rate of metabolic heat production. Incidentally, the rate at which exercising muscle breaks down its glycogen and releases lactate is exacerbated in hot environments (Fink et al., 1975; Febbraio et al., 1994; Parkin et al., 1999; Hargreaves, 2008); the mechanisms responsible are thought to include increased muscle temperatures and levels of circulating epinephrine (Hargreaves, 2008). Epinephrine is among the primary hormonal mediators of the stress response, and the dynamic regulation of its secretion is a central example of allostasis (McEwen, 2000). Allostasis, a term coined by Sterling and Eyer (1988) to refer to physiologic stability through variation, has been used to describe the brain-body involvement in budgeting resources in anticipation of biologic needs. Thus, allostasis serves homeostasis, a key parameter of which is stable core body temperature in homeotherms. If the release (e.g., muscle) and consumption (e.g., brain) of lactate during exercise in hot environments constitutes an allostatic means of anticipating and responding to thermal stress cues, then one would expect this to be reflected in training adaptations associated with acclimation to exercise in hot environments. Indeed, it is.

A decrease in metabolic heat production has been observed with acclimation to exercise in a hot environment (e.g., Sawka et al., 1983; Périard et al., 2015; Rivas et al., 2017). While the observed effects on metabolic heat production varies, additional metabolic adaptations may reflect the type of unevenly balanced metabolic heat production strategy hypothesized in the current work. These include a relatively small decrease in muscle glycogenolysis, and attenuated lactate accumulation (Périard et al., 2015). Because the typical increases in lactate threshold observed with endurance exercise training are thought to be more a result of augmented rates of lactate clearance (Donovan and Brooks, 1983; Donovan and Pagliassotti, 1989, 1990; MacRae et al., 1992; Phillips et al., 1995; Bergman et al., 1999; Messonnier et al., 2013), as opposed to its release, this suggests that acclimation to exercise in hot environments increases the rates of lactate clearance from circulation, in line with a metabolic thermoregulatory function for mitochondrial lactate oxidation proposed in the current work. Whether this is true, and if so, important, will require further research. It should also be noted that the most effective strategy to mitigate the hyperthermic effects of exercise in hot environments appears to be aerobic fitness itself (Alhadad et al., 2019), the development of which is well associated with increases in lactate threshold, and thus rate of lactate clearance.

The therapeutic use of intravenous (IV) infusion of lactate for type 2 diabetes, Alzheimer’s disease, cardiac disease, and traumatic brain injury (TBI) was recently reviewed (van Gemert et al., 2022). Of these, TBI patients, in particular, appear to benefit from IV lactate (Bouzat et al., 2013; Bouzat and Oddo, 2014; Gantner et al., 2014; Brooks and Martin, 2015; Carpenter et al., 2015). The mechanisms by which IV lactate improves long-term outcomes in patients with TBI are not fully understood, but it is interesting to note that systemic hyperthermia is a common occurrence after TBI and may even induce secondary brain injury (Svedung Wettervik et al., 2021). In light of the results of the current work, it is tempting to speculate about a role for IV lactate in mitigating the effects of hyperthermia associated with TBI due to the “cooler” thermo-neuroenergetics of lactate oxidation.

The export of lactate has been shown to play a vital role in tissues which require rapid activation such as fast-twitch glycolytic muscles or neurons following activation and neurotransmitter release vis-a-vis the Lactate Efflux model (Shulman et al., 2001). In both cases, glycolysis is used to meet transient spikes in energy demand. However, the TCA cycle is not initiated even in the presence of excess oxygen. A related phenomenon is observed in many cancer cells, which display an increased uptake of glucose and fermentation to lactate even in the presence of functional mitochondria and sufficient oxygen levels (i.e., the Warburg effect) (Liberti and Locasale, 2016). A recent investigation aimed to explore the Warburg effect from the perspective of metabolic thermogenesis. Using ^13^C-metabolic flux analysis of 12 cultured cancer cell lines combined with *in silico* metabolic simulations, Okahashi et al. (2023) present evidence that point to a reduction in metabolic heat production as a potential explanation for the increased reliance on glycolytic metabolism in cancer. In light of the evidence for lactate shuttling in cancer cells (e.g., Sonveaux et al., 2008; Goodwin et al., 2015) and the results of the present work, mitigating metabolic heat via intercellular lactate and/or pyruvate (Hong et al., 2016) exchange and metabolism among cancer cells with elevated demands for ATP seems plausible. It is noted that whole-body and localized-regional hyperthermia has long been used as an effective cancer treatment alone, or as an adjuvant therapy to traditional cancer treatments (Wust et al., 2002; Mallory et al., 2016), though a complete understanding of the basic mechanisms by which heat achieves its therapeutic effects is lacking (Lee et al., 2020).

In conclusion, we have presented evidence suggesting that the rate of heat production in lactate-fed mitochondria is intriguingly low in the mammalian brain. This is in accordance with a hypothetical working model in which the lactate shuttle serves a thermoregulatory function in the brain (Figure 1). To what extent this may contribute to the integrative thermoregulation of the intact organism awaits further investigation.

## Data availability statement

The raw data supporting the conclusions of this article will be made available by the authors, without undue reservation.

## Ethics statement

The animal study was reviewed and approved by St. Francis Xavier University Animal Care Committee.

## Author contributions

DK, EN, and DM contributed to conception and design of the study. DK, AF, EN, KB, and DM were involved in data collection. DK, EN, and AF analyzed data. DK, AF and DM interpreted the results. DK and AF wrote the manuscript. KB and DM contributed to revisions. All authors contributed to the article and approved the submitted version.

## Funding

This work was supported by NSERC Discovery Grant RGPIN-2015-04286 (DK) and Research NS Scotia Scholars 2022-2111 (EN).

## Conflict of interest

The authors declare that the research was conducted in the absence of any commercial or financial relationships that could be construed as a potential conflict of interest.

## Publisher’s note

All claims expressed in this article are solely those of the authors and do not necessarily represent those of their affiliated organizations, or those of the publisher, the editors and the reviewers. Any product that may be evaluated in this article, or claim that may be made by its manufacturer, is not guaranteed or endorsed by the publisher.
